# Lipopolysaccharide increases the release of VEGF-C that enhances cell motility and promotes lymphangiogenesis and lymphatic metastasis through the TLR4- NF-κB/JNK pathways in colorectal cancer

**DOI:** 10.18632/oncotarget.12449

**Published:** 2016-10-04

**Authors:** Guangwei Zhu, Qiang Huang, Yongjian Huang, Wei Zheng, Jin Hua, Shugang Yang, Jinfu Zhuang, Jinzhou Wang, Jianxin Ye

**Affiliations:** ^1^ Department of Gastrointestinal Surgery 2 Section, The First Hospital Affiliated to Fujian Medical University, Fuzhou, Fujian, China; ^2^ Key Laboratory of Ministry of Education for Gastrointestinal Cancer, Fujian Medical University, Fuzhou, Fujian, China

**Keywords:** lipopolysaccharide, colorectal cancer, VEGF-C, cell motility, lymphatic metastasis

## Abstract

Lipopolysaccharide (LPS) exists in the outer membrane of Gram-negative bacteria. Colorectal normal epithelium and colorectal cancer cells *in situ* are continuously exposed to LPS from intestinal bacteria, while little is known about the influence of LPS on colorectal cancer progression and metastasis. In this study, we investigated the potential role of LPS on colorectal cancer progression and metastasis as well as the underlying mechanisms. We measured higher LPS concentration in colorectal cancer tissues and even higher LPS concentration in colorectal cancer tissues with lymph node metastasis. LPS significantly enhanced cancer cell motility and promoted human dermal lymphatic endothelial cells' (HDLECs') capacity of tube-like formation *in vitro*, as well as accelerates lymphangiogenesis and lymph node metastasis in nude mice. Furthermore, we demonstrated LPS notably increased the expression of VEGF-C in a time-dependent and concentration-dependent manner. VEGF-C is a key regulator for lymphangiogenesis and lymph node metastasis. By constructing lentivirus-mediated shVEGF-C cells, VEGF-C down-regulation suppressed LPS' promotive effect on cancer cell motility and HDLEC tube-like formation capacity. In addition, we found TLR4- NF-κB/JNK signal pathways were important for LPS to increase VEGF-C expression. All these result suggested a critical role for LPS in migration, invasion, lymphangiogenesis and lymph node metastasis of colorectal cancer, providing evidence that LPS increased VEGF-C secretion to promote cell motility and lymphangiogenesis via TLR4- NF-κB/JNK signaling.

## INTRODUCTION

Colorectal cancer (CRC) is the fourth leading cause of cancer-related death in the world and the fifth leading cause in China, and the incidence and mortality have a gradually increasing trend [1, 2]. Long-term microbial infection may cause colon mucosa metaplasia, atypical hyperplasia and carcinoma *in situ*, finally leading to colon cancer. Subsequently, the microbial infection may promote colon cancer metastasis. Bacteria is an important component of the human body, while dysbiotic microbiota may promote disease development and contributes to the progression of colorectal cancer [3, 4]. Previous studies suggested that bacterial infection can activate immune cells to recognize tumor antigen which can cause immune reactions to kill tumor cells [5, 6], but long-term infection can raise inflammatory factors and cells to promote tumor progression and metastasis [7–9]. Recent experimental and clinical data showed that besides the inflammatory cytokines, bacteria and their products are also important factors to promote tumor progression and metastasis [10, 11]. But the pathogenic mechanism is complex and unclear. So exploring the mechanism of intestinal flora promoting colon cancer metastasis, especially the mechanism of lymphatic metastasis [12] and designing effective drug treatment is of great significance to improve survival rates of patients with colorectal cancer.

In colorectal cancer, the intestinal microbiota and their metabolic microenvironment are usually altered and a significantly increased diversity of Gram-negative bacteria subgroups was noted [13, 14]. LPS is known as the wall of Gram-negative bacteria. We speculated that LPS is a key factor of bacterial infection in colorectal cancer progression and metastasis. Increased Gram-negative bacteria infection leads to greater release of LPS in colorectal tumor *in situ*. Previous studies reported that LPS can promote epithelial-mesenchymal transition, cell migration and invasion through NF-kB-Snail signaling [15–17]. LPS can increase lymphangiogenesis involving with VEGF-C, VEGFR-3 and pro-inflammatory cytokines [18, 19]. These studies suggested that LPS mainly contributes to tumor metastasis by accelerating cell motility and promoting lymphangiogenesis. While past efforts only demonstrated the mechanism of LPS-induced cell motility, whether LPS contributed to lymph node metastasis and especially which signal was affected remains obscure. Therefore, we focused on the role of LPS in lymphatic metastasis and tried to find out whether LPS upregulates cell motility and lymphangiogenesis to further promote lymphatic metastasis.

For lymphatic metastasis, VEGF-C and VEGFR3 are key factors. VEGF-C and VEGFR3 are prominent in regulating the lymphatic vasculature [20, 21]. VEGF-C is involved in lymphangiogenesis and contributes to cell migration and invasion leading to poorer prognosis of colorectal cancer [22–24]. In macrophages, LPS triggers the expression of VEGF-C and VEGFR3 by TLR4-NF-kB signaling [25]. In our previous study, we found LPS can also increase VEGF-C expression in colorectal cancer cells. However, the molecular mechanisms of LPS on VEGF-C/VEGFR3 expression up-regulation remain unclear in colorectal cancer.

Therefore, to examine whether LPS is involved in lymphatic metastasis, we measured LPS concentration in human colorectal tissues. Besides, we identify the roles of LPS in VEGF-C expression up-regulation, cell motility, lymphangiogenesis *in vivo* and *in vitro* experiments. We assumed that LPS increased the expression of VEGF-C to promote cell motility, lymphangiogenesis and lymphatic metastasis in colorectal cancer.

## RESULTS

### LPS concentration in colorectal cancer tissues and normal mucosa

**Figure 1:**
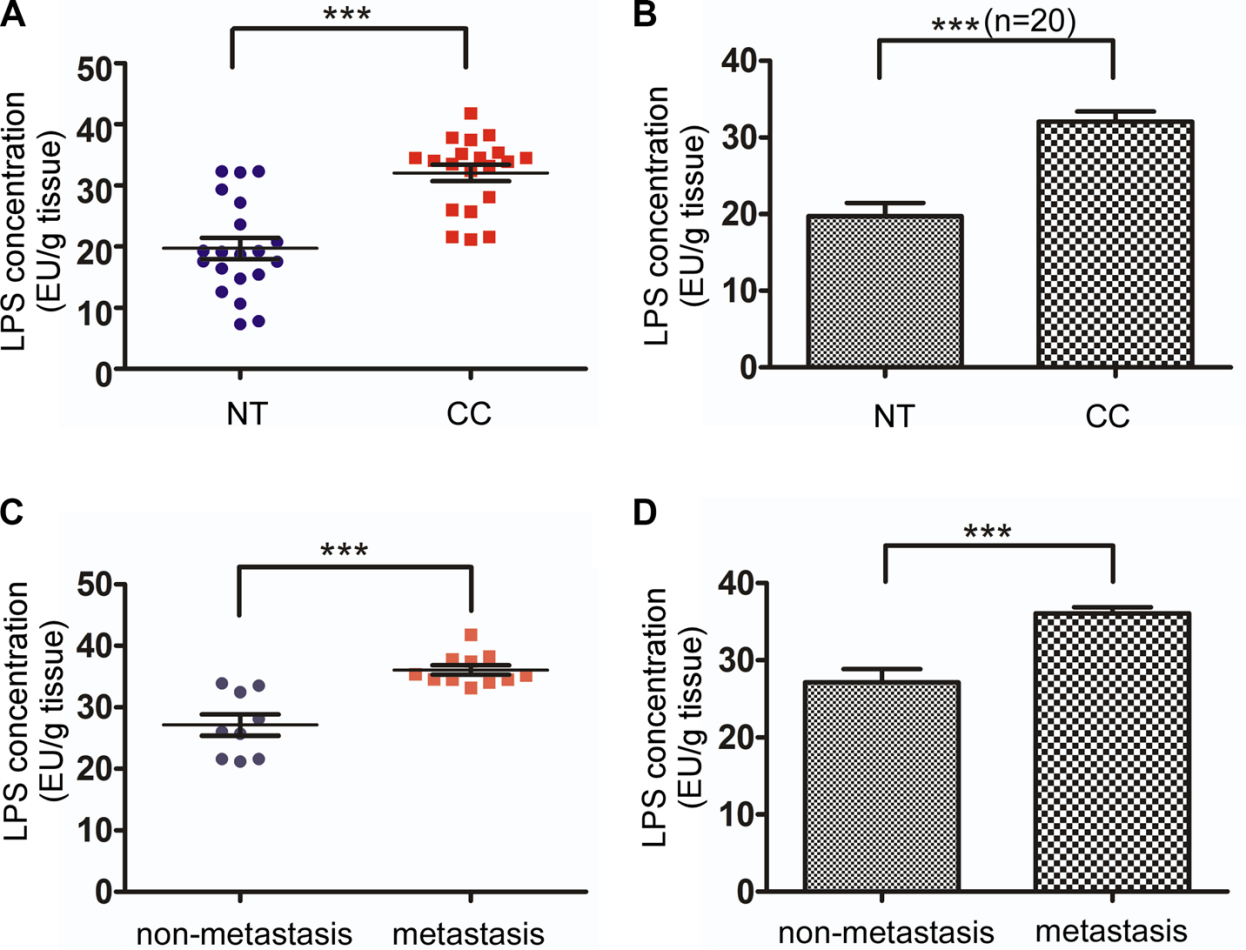
LPS concentration in colorectal cancer tissues and normal mucosa (**A**) LPS concentration was significantly higher in 20 colorectal cancer tissues compared with matched normal tissues. (**B**) Average LPS concentration in 20 colorectal cancer tissues and matched normal tissues. (**C**) Lymph node metastasis (*n* = 11) and none lymph node metastasis (*n* = 9) colorectal tissue LPS concentration. (**D**) Average LPS concentration of Lymph node metastasis and none lymph node metastasis colorectal tissues. Expression was shown for LPS quantity in 1 gram colorectal tissue (EU: endotoxin unit).

To measure the LPS concentration in colorectal cancer tissues and corresponding normal mucosa, we used Tachypleus amebocyte lysate endotoxin detection assay for 20 pairs of specimens. These specimens all got the patients' permission. The patients comprise 11 men and 9 women, whose ages ranged from 35 to 70, with an average of 61 years. Pathological stages by TNM classification and case numbers were as follows: 2 cases of pI, 7 cases of pII, 10 cases of pIII and 1 cases of pIV. In normal mucosa, LPS concentration was low (19.719 ± 7.708, mean ± standard deviation, Figure 1A and 1B). In contrast, LPS concentration was much higher in colorectal cancer tissues (32.047 ± 5.966, mean ± standard deviation, Figure 1A and 1B). There was significant difference between colorectal cancer tissues and corresponding normal mucosa (*p <* 0.0001). Then we divided colorectal cancer tissues into lymph node metastasis group and no lymph node metastasis group. After analysis we noticed lymphatic metastasis group LPS concentration (36.075 ± 2.533, mean ± standard deviation, Figure 1C and 1D) was significantly higher than no lymph node metastasis group (27.125 ± 5.192, mean ± standard deviation, Figure 1C and 1D). Detailed data was shown in Supplementary Table S1 and S2.

### LPS treatment increases VEGF-C expression in colorectal cells

**Figure 2:**
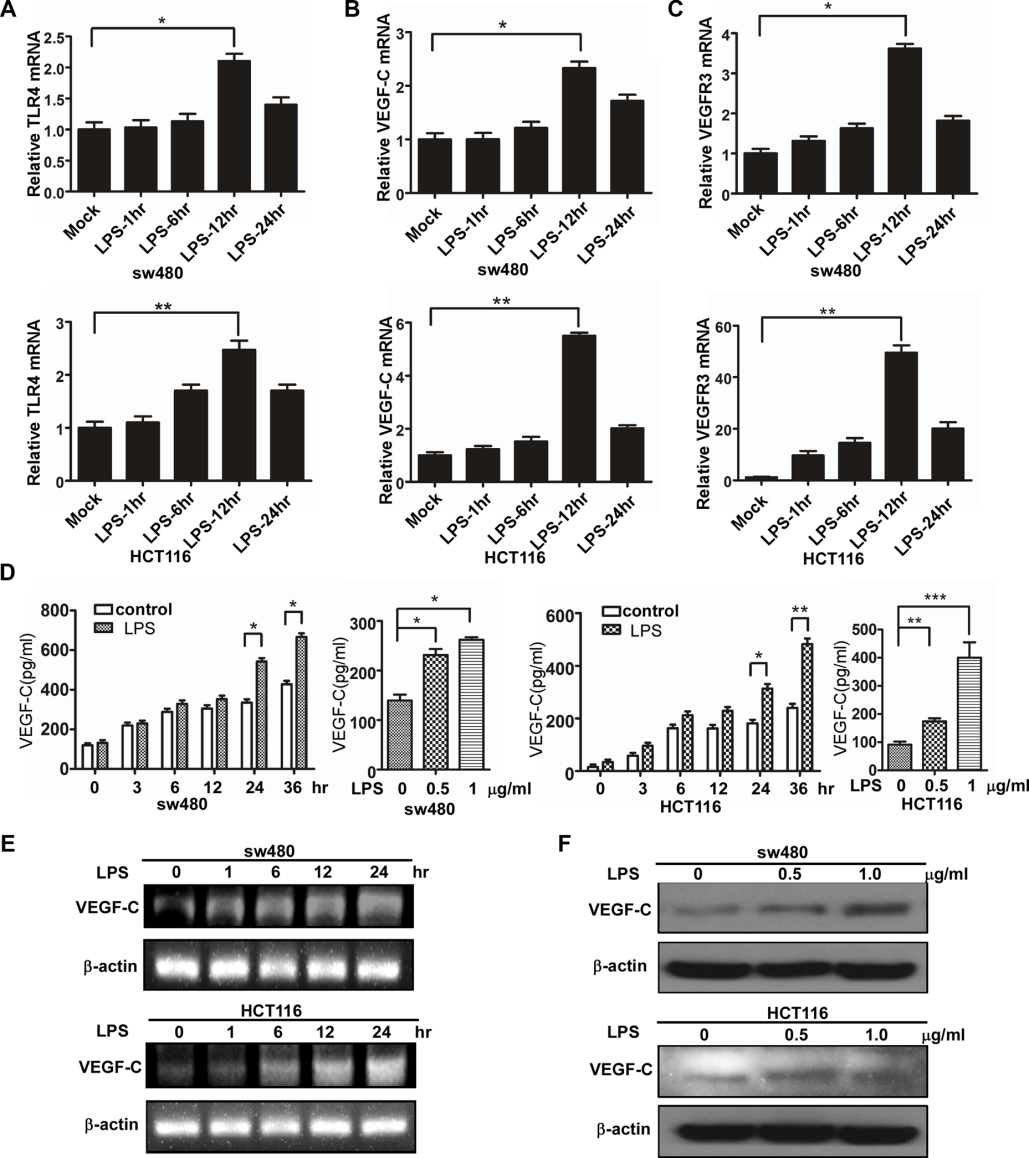
LPS treatment enhances VEGF-C expression in colorectal cancer cells (**A**–**C**) The mRNA of TLR4, VEGF-C and VEGFR3 in the mock, LPS-stimulated (1 μg/ml) sw480 and Hct116 colorectal cells by real-time PCR. (**D**) The protein expression of VEGF-C from the mock, LPS-stimulated sw480 and Hct116 colorectal cells by ELISA. (**E**) VEGF-C mRNA expression in the mock, LPS-stimulated (1 μg/ml) sw480 and Hct116 cells by agarose gel electrophoresis. (**F**) The protein expression of VEGF-C from the mock, LPS-stimulated sw480 and Hct116 colorectal cells by western blot. Error bars represent mean ± SEM, representative of three experiments, *p < %0.05, **p < %0.01, ***p < %0.001.

To identify relevant mRNA changes, real-time PCR assay was performed to detected TLR4, VEGF-C and VEGFR3 expression after LPS treatment (1 μg/ml) at various time points. As shown in Figure 2A–2C, the mRNA expression of TLR4, VEGF-C and VEGFR3 increased in a time-dependent manner in sw480 and Hct116 cells. And agarose gel electrophoresis was consistent with the results (Figure 2E). To identify relevant protein changes, ELISA analysis showed that secreted VEGF-C protein was also increased in a time-dependent and dose-dependent manner in sw480 and Hct116 cells (Figure 2D). And western blot was consistent with the results (Figure 2F).

**Figure 3:**
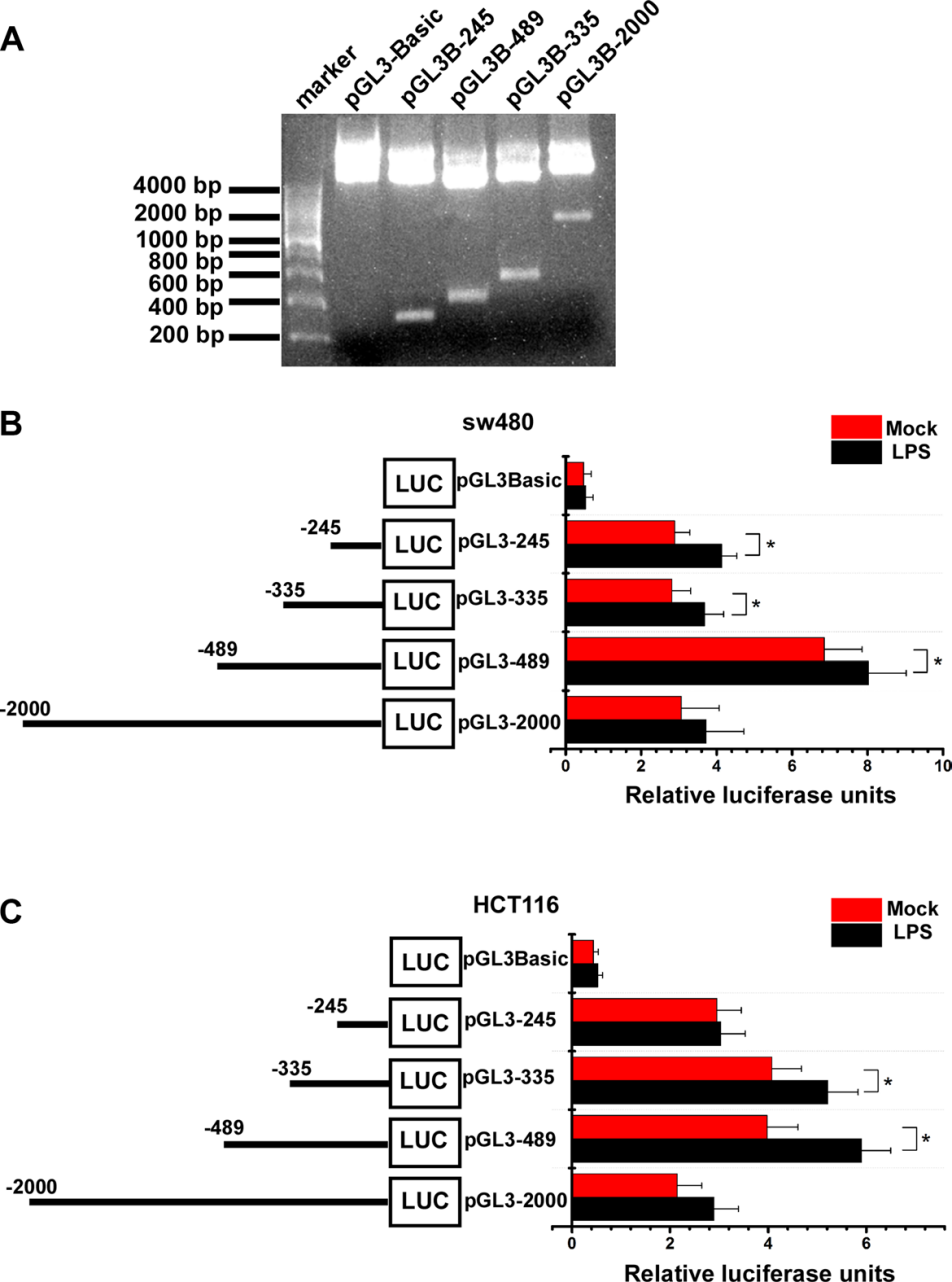
Activity analysis of VEGF-C promoter (**A**) the full length promoter and various promoter deletions of VEGF-C. (**B** and **C**) Mock and LPS-stimulated (1 μg/ml) colorectal cancer cells were transfected with 250 ng of each VEGF-C promoter construct (reporter plasmid); 40 ng of the renilla luciferase expression vector pRL-TK was used for normalization, and the promoterless vector pGL3-Basic served as the negative control. Luciferase activities were measured 36–48 hours after transfection and the relative luciferase units (RLU) were obtained by normalization with renilla luciferase. Error bars represent mean ± SEM, representative of three experiments, *p < %0.05.

To further identify LPS' effect on VEGF-C expression, we construct VEGF-C full length promoter and various VEGF-C promoter deletions (Figure 3A). Full length and a series of deletion constructs of the VEGF-C promoters were transfected transiently into the sw480 and HCT116 colorectal cancer cells. Dual-luciferase reporter assay was used to detect VEGF-C expression of control group and LPS-treated group (1 μg/ml). Relative luciferase unit increased with the length of VEGF-C promoter extending, but declined for the full length promoter. This phenomenon may result from negative regulatory element which exits in the front region of the full length promoter.

For sw480, more relative luciferase units were detected in LPS treated groups, significantly in pGL3-245, pGL3-335 and pGL3-489 (Figure 3B). For Hct116, more relative luciferase units were detected in LPS treated groups, significantly in pGL3-335 and pGL3-489 (Figure 3C).

These results indicated that LPS treatment increases VEGF-C expression in a time-dependent and concentration-dependent manner.

### LPS enhances colorectal cancer cell motility but proliferation is not increased

**Figure 4:**
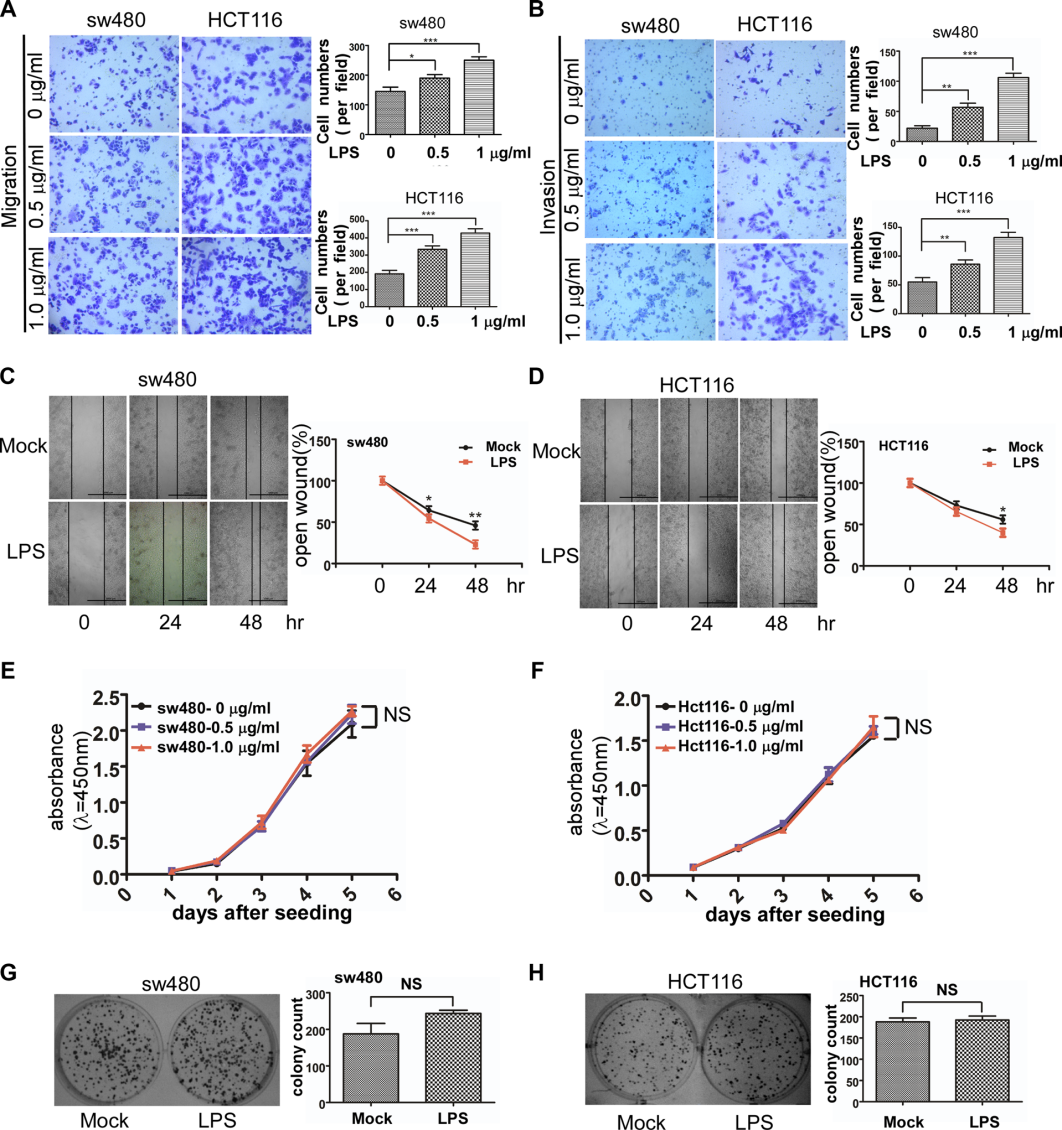
Effects of LPS on cell motility and proliferation of sw480 and Hct116 cells (**A** and **B**) Representative images of migrated and invaded colorectal cancer cells through chambers' membrane (100×) treated with LPS ( 0 , 0.5 , 1 μg/ml). Cell numbers were counted in three randomly selected microscopic fields. (**C** and **D**) Representative images of mock and LPS-stimulated colorectal cancer cell wound healing (40×). Microscopic observations were photographed 0, 24 and 48 hours after scratching the cell surface. (**E** and **F**) Effects of LPS (0 , 0.5 , 1 μg/ml) on cell growth by CCK8 assay. (**G** and **H**) Effects of LPS (1 μg/ml) on cell growth by colony formation assay. Error bars represent mean ± SEM, representative of three experiments, *p < %0.05 , **p < %0.01, ***p < %0.001, NS *p >* 0.05. Scale bars represent 1000 μM.

We used a transwell assay to observe colorectal cancer cell motility change after LPS stimulation. Migration and invasion ability of both sw480 and Hct116 cells increased with LPS (0, 0.5, 1 μg/ml) treatment (Figure 4A and 4B). There was statistical significance among different groups (*p <* 0.05). To further verify the effects of LPS on cell motility, we performed wound healing assays which showed LPS enhanced sw480 cell migration ability in a time-dependent alteration (Figure 4C). To confirm this result, the same experiment was performed in the Hct116 cells (Figure 4D).

Then we investigated whether LPS affected colorectal cancer cell proliferation. CCK-8 assay was used to observe sw480 and Hct116 growth treated with LPS (0, 0.5, 1 μg/ml) in 5 days. As shown in Figure 4E and 4F, LPS has no effect on cell proliferation. We also performed colony-formation assay which also showed no significance (Figure 4G and 4H).

These results indicated that LPS improved sw480 and Hct116 colorectal cancer cell motility but had little effect on cell proliferation.

### VEGF-C is critical in LPS induced cell motility enhancement

**Figure 5:**
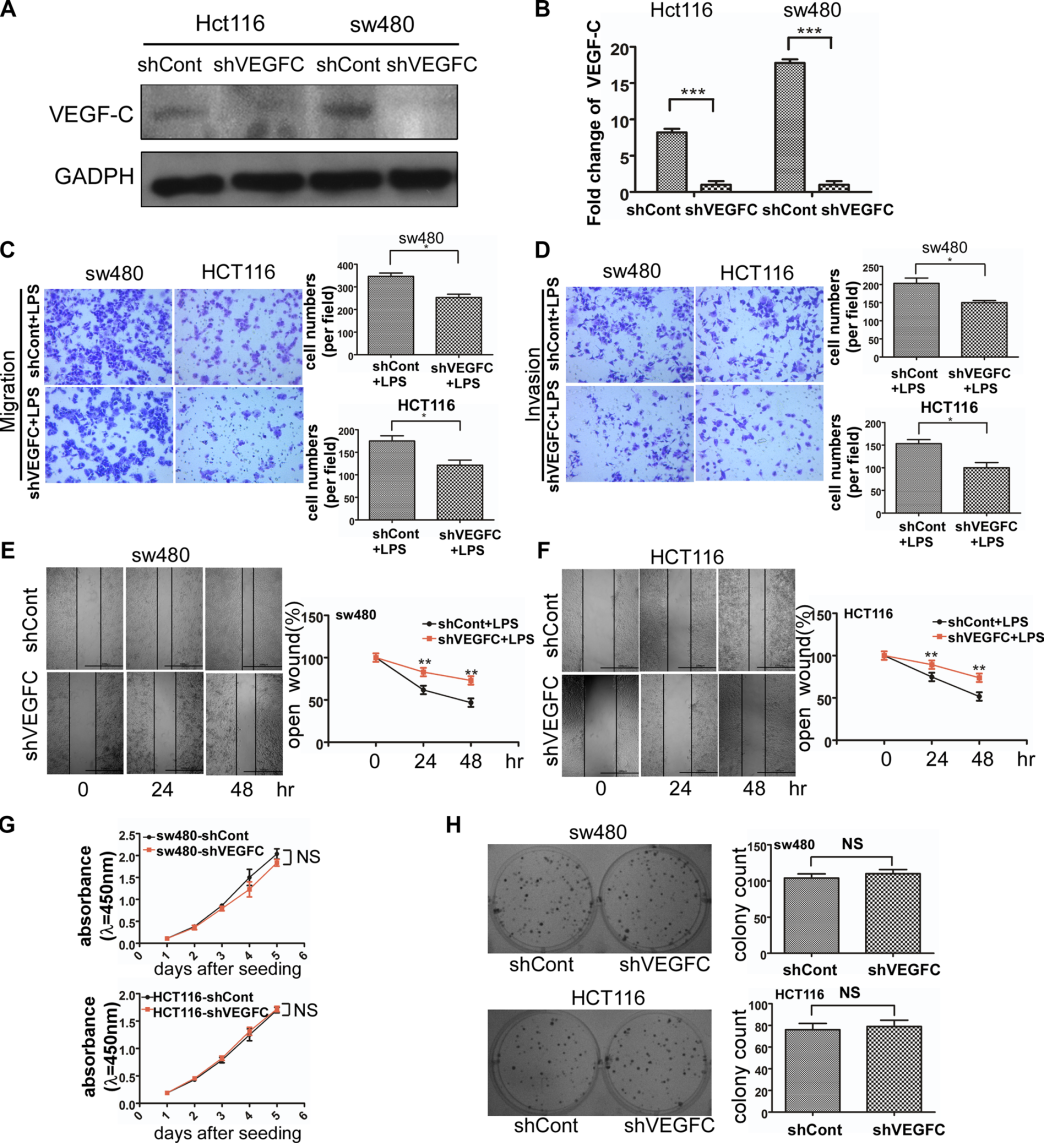
Effects of VEGF-C on cell motility and proliferation on sw480 and Hct116 cells (**A**) Western blot analysis of identifying VEGF-C expression in VEGF-C knockdown sw480 and Hct116. The quantified analysis of the expression of VEGF-C has been presented on (**B**). (**C** and **D**) Representative images of migrated and invaded colorectal cancer cells through chambers' membrane (100×). Cell numbers were counted in three randomly selected microscopic fields. (**E** and **F**) Representative images of LPS stimulated shControl and shVEGFC colorectal cancer cell wound healing (40×). Microscopic observations were photographed 0, 24 and 48 hours after scratching the cell surface. (**G**) Effects of VEGF-C on cell growth by CCK8 assay. (**H**) Effects of VEGF-C on cell growth by colony formation assay. Error bars represent mean ± SEM, representative of three experiments, *p < %0.05, **p < %0.01, ***p < %0.001, NS *p >* 0.05. Scale bars represent 1000 μm.

To identify the role of VEGF-C in colorectal cancer cell motility enhancement, we constructed VEGF-C knockdown stable cell line. We selected the most effective shRNA pair from 4 pairs of shRNA for our subsequent experiment. The effect of VEGF-C silencing was identified by western blot for sw480 and Hct116 colorectal cancer cells (Figure 5A).

We used transwell migration and invasion assays to examine the cell motility of sw480 and Hct116 colorectal cancer cells. We observed that VEGF-C down-regulation decreased the migration and invasion ability of sw480 and Hct116 cells (Supplementary Figure S1A).

In order to confirm the role of VEGF-C in LPS induced cell motility enhancement, we performed a transwell assay. As shown in Figure 5C and 5D, VEGF-C down-regulation suppressed LPS induced cell motility. And wound healing assay was consistent with the result (Figure 5E and 5F). These results indicated that LPS enhanced colorectal cancer cell motility was partially caused by VEGF-C.

In addition we also managed CCK-8 assay and colony formation assay to explore sw480 and Hct116 cell proliferation alteration of aberrant expression of VEGF-C. Our result showed no statistical significance between control group and shVEGF-C group (Figure 5G and 5H).

### LPS promotes lymphangiogenesis and lymph node metastasis via VEGF-C *in vitro* and *in vivo*

**Figure 6:**
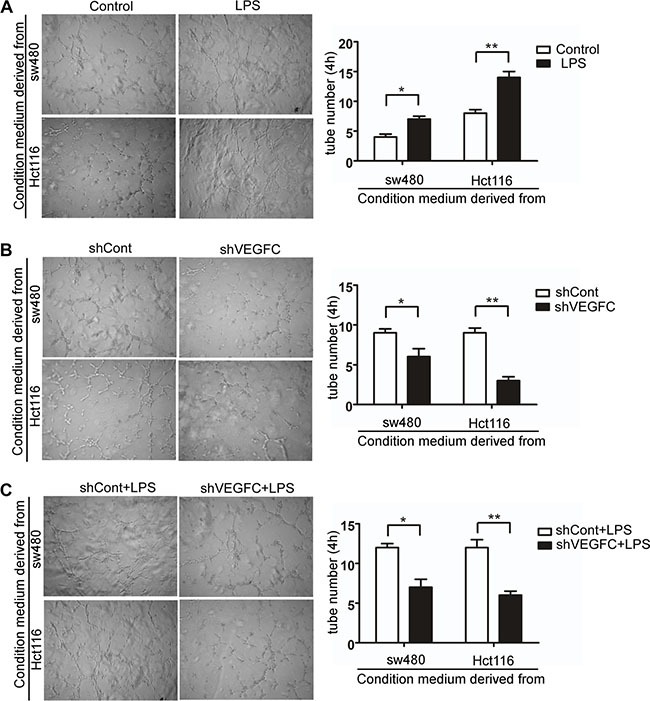
LPS promotes colorectal cancer lymphangiogenesis via VEGF-C *in vitro* (**A**–**C**) Representative images and quantitative results of human dermal lymphatic endothelial cells (HDLECs) cultured with conditioned medium derived from LPS-treated (1 μg/ml) cells and control cells, VEGF-C silenced cells and control cells, VEGF-C silenced cells treated with LPS (1 μg/ml) and control cells treated with LPS (1 μg/ml) (40×). Tube numbers were counted in three randomly selected microscopic fields. Error bars represent mean ± SEM, representative of three experiments, *p < %0.05, **p < %0.01.

In the present study, we performed an indirect co-culture system to evaluate the role of LPS in lymphangiogenesis *in vitro*. We evaluated the tubule formation capacity of HDLECs, which is an essential step for lymphangiogenesis. As shown in Figure 6A, after co-cultured with LPS treated colorectal cells' supernatants over 4 hours, the tubule-like structure formation capacity of HDLECs increased compared with control group in sw480 and Hct116 (*p <* 0.05 and *p <* 0.01). And silencing VEGF-C significantly decreased HDLECs' capacity of tubule-like structure formation (Figure 6B). As show in Figure 6C, VEGFC down-regulation suppressed LPS stimulated tubule formation capacity of HDLECs. These results indicated that LPS promotes lymphangiogenesis via VEGF-C.

**Figure 7:**
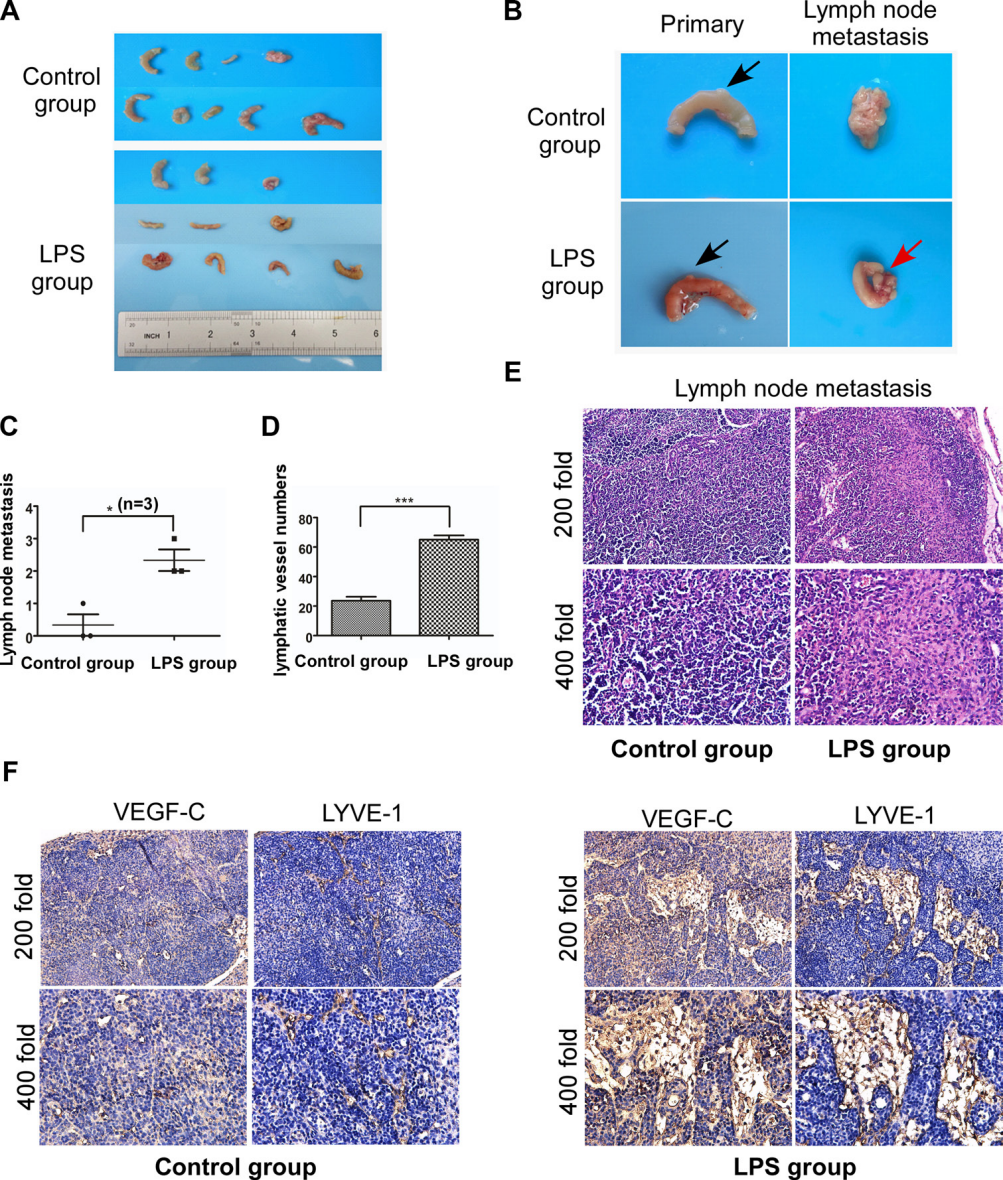
LPS promotes lymphangiogenesis and lymph node metastasis via VEGF-C *in vivo* (**A** and **B**) Mice were sacrificed and primary tumor (black arrow) and metastatic lymph node (red arrow) were collected. (**C**) Numbers of lymph node metastasis for control group and LPS treated group. (**D**) Lymphatic vessel density of metastatic lymph node (LPS group) and normal lymph node (control group). (**E**) Metastatic and normal lymph nodes H-E staining. (**F**) Metastatic and normal lymph nodes staining with antibodies to VEGF-C and LYVE-1. Error bars represent mean ± SEM, representative of three experiments, ***p < %0.001.

On the basis *in vitro*, we investigated whether LPS can influence lymphangiogenesis and lymph node metastasis by establishing mice models of colorectal cancer. The detailed processes were shown in Supplementary Figure S1. Two months later, mice were sacrificed and primary tumors or metastatic lymph nodes were collected and counted (Figure 7A and 7B). Metastatic lymph nodes were further confirmed by HE staining (Figure 7E). There was no significant difference in primary tumor diameter and numbers between LPS group and control group, while more lymph node metastasis were observed in LPS group (Figure 7C, *p <* 0.05). We then performed VEGF-C and LYVE-1 immunohistochemistry. As shown in Figure 7F, VEGF-C expression increased in LPS treated metastatic lymph node, but faint in matched normal lymph node. And LPS increased the Lymphatic vessel density of metastatic lymph node in LPS treatment group (Figure 7F). The detailed lymphatic vessels numbers were described in Figure 7D, and there was significant difference between LPS group and control group (*p <* 0.001).

Our data *in vivo* was consistent with the results *in vitro*, demonstrating that LPS promote lymphangiogenesis and lymphatic metastasis via VEGF-C.

### TLR4-NF-ΚB/JNK signal pathways trigger VEGF-C expression

**Figure 8:**
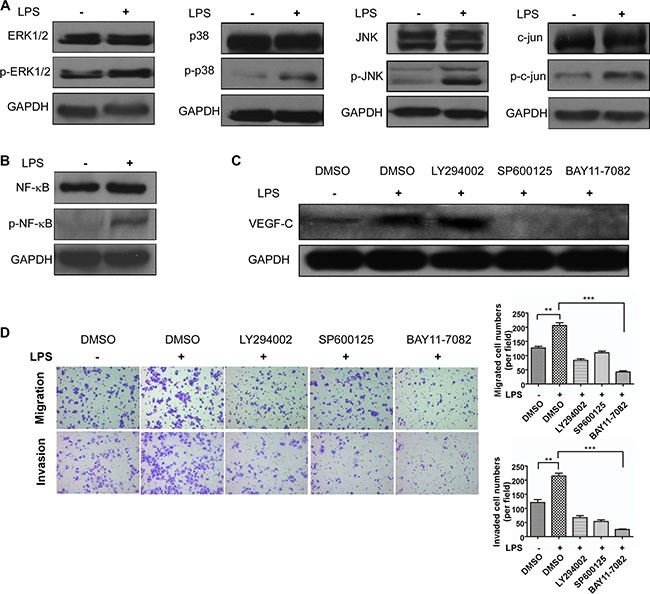
TLR4-NF-κB/JNK signal pathways trigger VEGF-C expression (**A**) Western blot analysis of ERK1/2, p38, JNK and c-jun expression. (**B**) Western blot analysis of NF-κB expression. (**C**) VEGF-C expression after LPS (1 μg/ml), DMSO (1 μl), LY294002 (20 μM), SP6001250 (10 μM) and BAY 11-7082 (12.5 μM) treatment. (**D**) LY294002 (20 μM), SP6001250 (10 μM) and BAY 11-7082 (12.5 μM) inhibit LPS (1 μg/ml) induced cell motility. Representative images of migrated and invaded colorectal cancer cells through chambers' membrane (100×). Cell numbers were counted in three randomly selected microscopic fields. Error bars represent mean ± SEM, representative of three experiments, **p < %0.01, ***p < %0.001.

Based on the effect of LPS increasing VEGF-C expression, we then investigated how VEGF-C expression was regulated. We stimulated sw480 cells with LPS (1 μg/ml, 12 hour) and performed western blot to detect TLR4 downstream signal pathways protein change. As shown in Figure 8A and 8B, mitogen-activated protein kinase (MAPK) family related protein, such as ERK1/2, p38, JNK, c-jun were activated. And phosphorylated NF-κB expression was also increased. Therefore we used JNK inhibitor SP600125 (10 μM) and NF-κB inhibitor BAY 11-7082 (12.5 μM) to treat sw480 cells for 24 hours and DMSO as the control. On the basis that NF-κB can be activated by AKT, we also used AKT inhibitor LY294002 (20 μM). Western blot showed NF-κB inhibitor and JNK inhibitor can decrease VEGF-C expression, however AKT inhibitor had no effect (Figure 8C).

In present study, we demonstrated VEGF-C expression can be regulated by two signal pathways. Then we explored how these inhibitors influenced colorectal cell motility. Transwell migration and invasion assay were managed, indicating that the two inhibitors can attenuate LPS induced cell motility (Figure 8D). These results demonstrated that LPS triggered VEGF-C expression via TLR4-NF-κB/JNK signal pathways.

## DISCUSSION

In our study, we measured LPS concentration of 20 pairs of colorectal cancer and corresponding normal mucosa tissues, demonstrating LPS concentration was high in colorectal cancer tissues and even higher in colorectal cancer tissues with lymphatic metastasis. Additionally, LPS can increase the expression of VEGF-C, thus accelerating cancer cell motility, lymphangiogenesis, and promoting lymphatic metastasis. The underlying mechanisms were associated with the activation of NF-κB and JNK.

Cck8 assay and colony formation assay showed that all the concentrations of LPS in the present study did not affect the viability of colorectal cancer cells, furthermore suggesting that the transwell assay of LPS accelerating cell motility were not due to cell proliferation. Whereas previous studies reported that LPS can increase tumor cell proliferation [26–28]. These results might result from different LPS source strain or cell particularity.

LPS is a common inflammogen that comes from the outer membrane of Gram-negative bacteria. Accumulating evidences have shown LPS can enhance lymphatic invasion [29] and are associated with lymphatic metastasis [26]. In our experiment, we found colorectal cancer tissues have higher concentration of LPS and even higher concentration of LPS in lymph node metastatic cancer tissues. We speculated that ischemic necrosis and bacterial infections often occurred in colorectal cancer tissues with high concentration of LPS. And LPS further promoted lymphatic metastasis. These data directly demonstrated the lymph node metastatic role of LPS, highly provided a possibility for new therapies on decreasing LPS to inhibit lymph node metastasis of colorectal cancer.

In the present report, we found LPS enhanced the motility of colorectal cancer cells. Previous studies have reported that LPS can induce epithelial-mesenchymal transition and promote cell adhesion and invasion [17, 30–32]. VEGF-C promoted cell migration through transcription of CNTN-1 [33] and promote metastasis via up-regulation and activation of RhoA/ROCK-2/moesin cascade [34]. We herein demonstrated that LPS increased cell migration and invasion via VEGF-C. We observed TLR4, VEGF-C, VEGFR-3 expression significantly increased after LPS treatment. TLR4 recognized LPS and activated downstream proteins to increase VEGF-C expression, then VEGFR-3 recognized VEGF-C and played the following biological functions. In our experiment, VEGFC down-regulation can suppress LPS induced cell migration and invasion enhancement. All these results indicated LPS-induced cell migration and invasion was partially caused by up-regulating expression of VEGF-C.

VEGF-C functions specifically to induce lymphangiogenesis and was related with prognostic evaluation in colon cancer patients [35–37]. Lymphangiogenesis is a primary cause for lymph node metastasis [38]. We demonstrated LPS increased HDLECs' capacity of tubule-like structure formation, which can be suppressed by VEGF-C down-regulation. And in LPS treated nude mice group, there was more VEGF-C protein expression in metastatic lymph node with more lymphatic vessels. These results proved that LPS promoted VEGF-C expression up-regulation to further affect lymphangiogenesis and lymph node metastasis.

Mechanism underlying elevated VEGF-C expression with LPS stimulation involves activation of NF-κB in macrophages [25]. Our studies indicated that ERK1/2, p38, JNK, NF-κB, AP-1 can be activated by LPS treatment. Therefore we used JNK inhibitor, NF-κB inhibitor and AKT inhibitor to treat LPS-stimulated colorectal cancer cells. We perceived that either NF-κB or JNK pathway inhibition can significantly decrease VEGF-C expression. LPS activates TLR4-MyD88-NF-kB signaling to up-regulate VEGF-C [25], while how JNK pathway was affected in VEGF-C secretion remains largely unknown and need our future studies.

In conclusion, we demonstrated that LPS increased the VEGF-C expression to enhance cell motility, lymphangiogenesis and lymph node metastasis through TLR4-NF-κB/JNK pathways. Our results implied LPS closely associates with colorectal carcinoma progression, suggesting that properly decreasing LPS concentration in colorectal cancer might have potential clinical applications to alleviate lymphatic metastasis.

## MATERIALS AND METHODS

### Materials

Lipopolysaccharide (Escherichiacoli 055:B5), LY294002 (S1105), SP600125 (S1460) and BAY 11-7082 (S2913) were obtained from Sigma-Aldrich (St Louis, MO, USA). LPS was dissolved in PBS, while other chemicals were dissolved in DMSO (Sigma-Aldrich). RPMI 1640 with L-glutamine and fetal bovine serum were from GIBCO (Gaithersburg, MD, USA). Chromogenic end-point TAL Kit (CE80545) was purchased from Chinese Horseshoe Crab Reagent Manufactory company (Xiamen, China). Human VEGF-C ELISA Kit was purchased from Boster Biological Technology (Wuhan, China).

### Cell lines and clinical samples

The two colorectal cancer cell lines (sw480, HCT116) were obtained from cell bank of the Chinese Academy of Sciences (Shanghai, China). Both cell lines were cultured in RPMI 1640 with L-glutamine supplemented with 10% fetal bovine serum in 5% CO2. Human dermal lymphatic endothelial cells (HDLECs, Sciencell, San Diego, California, USA) were incubated in endothelial cell medium (Sciencell). Human colorectal cancer samples and their paired adjacent normal colorectal mucosa tissues were collected from the First Affiliated Hospital of Fujian Medical University (Fuzhou, China) in 2015.

Samples were immediately stored in liquid nitrogen after surgical resection. All the patients in our study did not received preoperative chemotherapy, radiotherapy, or biological treatment. We got all the patients' written informed consent of the samples and the study was approved by the Ethics Committee of the First Affiliated Hospital of Fujian Medical University No. 2016 [053].

### Tachypleus amebocyte lysate for endotoxin detection assay

Colorectal cancer and paired adjacent normal tissue samples were incised to the weight of 0.05 g each by analytical balance (ME104E, mettle Toledo). All the samples were homogenized in lysing matrix tubes (MP Biomedicals, Eschwege, Germany) filled with 1 millilitre water for bacterial endotoxin test in FastPrep-24 (MP Biomedicals, Eschwege, Germany). Endotoxin concentration of samples were determined with Chromogenic end-point TAL Kit (CE80545, Xiamen, China) according to the manufacturer's instructions.

### RNA extraction and reverse transcription real-time quantitative PCR

Total RNA was extracted from cultured cells using Trizol reagent (ambion, Carisbad CA, USA) and RNA was reverse transcribed to cDNA using RT Reagent Kit (TaKaRa, Dalian, China). Then real-time quantitative PCR of cDNA was prepared using SYBR Premix EX Taq kit (Takara, Shiga, Japan) and amplification was performed on Mx3000P QPCR system (Agilent Technology, Santa Clara, CA, USA). Primers (TLR4, VEGF-C, VEGFR3) were used to detect the relative expression levels of the target genes by the 2^−ΔΔct^ method. The relative amount of target mRNA was normalized to β-actin. All the primers were designed by BioSune Biotechnology (Shang Hai) Co., Ltd.

### Enzyme-linked immunosorbent assay (ELISA)

Colorectal cancer cells were seeded in 24-well plates and incubated in RPMI 1640 with 10% FBS. The conditioned mediums were collected at different time points and human VEGF-C protein levels were quantified using the Quantitative analysis VEGF-C ELISA reagent (Boster Biological Technology, Wuhan, China) according to the manufacturer's instructions. The results presented represent the mean values from three separate experiments.

### Western blot analysis

Cells were lysed with Western & IP cell lysis buffer (Beyotime, Shanghai, China) containing PMSF (Amresco, Solon, Ohio, USA) on ice for 30 minutes, then the cell lysis solution was centrifuged at 12, 000 g for 10 min at 4°C and the supernatant was collected. Cell protein (60 μg per lane) was separated by 10% SDS-PAGE and transferred onto a 0.45 μM PYDF membrane (Amersham™Hybond™, Germany). The membrane was blocked with 0.5% bovine serum album (Amresco, Solon, Ohio, USA) at room temperature for 2 hours. Then the membrane was incubated with rabbit anti-VEGF-C (1:1, 000; Abcam, ab9546), rabbit anti-GAPDH (1:1, 500; Abcam, ab181602), rabbit anti-NF-κB p65 (1:1, 000;cell signaling, #8242), rabbit anti-Phospho- NF-κB p65 (1:1, 000; cell signaling, #3033), rabbit anti-p38 MAPK (1:2, 000; cell signaling, #8690), rabbit-anti-Phospho-p38 MAPK (1:2, 000; cell signaling, #4511), rabbit anti-ERK1/2 (1:2, 000; cell signaling, #4695), rabbit anti-phospho-ERK1/2 (1:2, 000; cell signaling, #4094), rabbit anti-JNK (1:1, 500; cell signaling, #9252), rabbit anti-phospho-JNK (1:1, 500; cell signaling, #4668), rabbit anti-c-jun (1:1, 500; abcam, ab32137), rabbit anti-phospho-c-jun (1:1, 500; abcam, ab32853), overnight at 4°C. The membranes were washed three times with TBS-T (0.1% Tween-20) for 10 minutes each at room temperature. Then the membranes were incubated in secondary antibody for 30 minutes at room temperature and washed three times. Subsequently the membranes were detected using enhanced chemiluminescence substrate detection solution (Lulong Biotech, Xiamen China).

### VEGF-C shRNA knockdown

VEGF-C gene knockdown was constructed by stable transduction with lentivirus, using the Psuper. retro. puro vector containing a VEGF-C specific shRNA. And a scrambled nucleotide sequence was used as the negative control. Stable cell lines were screened with puromycin and identified by western blotting.

### Cell proliferation assay

Cells were seeded onto 96-well plates at a density of 1, 500 cells per well and detected using the cell counting kit CCK-8 (Donjindo, Kumamoto, Japan) by a microplate reader (Bio-Tek, Winooski, VT, USA) for 5 days. The data of absorbance at a wavelength of 450 nm was collected to analyze cell proliferation.

### Colony formation assay

Cells were seeded onto 6-well plates at a density of 500 cells per well and cultured for 2 weeks. Then cells were fixed by methanol for 10 minutes and stained with crystal violet. Colonies of 50 or more cells were counted.

### Cell migration and invasion assay

Tranwell chamber (8 μM, 24-well format; FALCON) and Matrigel coated transwell chamber (BD Bioscience) were inserted into 24 well cell cuture plates to measure migration and invasion. 9 × 10^4^ cells in 0.3 mL of serum-free medium were added in the upper chamber, while 0 8 mL of RPMI 1640 containing 10% FBS was added to the lower chamber. For the pharmaceutical experiment with inhibitors, cells were incubated with LY294002 (20 μM), SP6001250 (10 μM) and BAY 11-7082 (12.5 μM) for 24 hours before proceeding with the transwell assay described above, the lower and upper chambers both contained inhibitors. Cells were cultured for 24 hours. Then cells with transwell chambers were fixed in methanol for 5 minutes and stained with crystal violet and counted in 3 random fields under microscope.

### Wound healing assay

Cells were seeded onto 6-well plates and cultured until convergence degree reached 100%. Wounds were scratched on the monolayer cells using 20 μl pipette tips. The plates were photographed at the time of 0 hour, 24 hours, 48 hours after cells seeding in FBS-free RPMI 1640.

### VEGF-C promoter luciferase construction

Genomic DNA was extracted from sw480 cells using TIANamp Genomic DNA Kit (Tiangen, Beijing, China) and used as a template for polymerase chain reaction (PCR) amplification. Full length VEGF-C promoter (pGL3B-2000, nucleotides −2000 to 0) and various VEGF-C promoter deletion including pGL3B-489 (nucleotides −489 to 0), pGL3B-335 (nucleotides −335 to 0), pGL3B-245 (nucleotides −245 to 0) were made by inserting the corresponding PCR-generated fragment into the Xhol and HindIII sites of the pGL3-Basic plasmid. The primers used are shown in Supplementary Table S3. The reverse primer 2000-245R was used with all the other forward primers.

### Dual-luciferase reporter assay

Cells were seeded into a 24 well plates and cotransfected with 250 ng reporter plasmid and 40 ng of the Renilla luciferase encoding plasmid pRL-TK after 36~48 hours incubation with PGL3B-basic as the control. Cells were lysed with passive lysis buffer, and the reporter activity was detected using the Dual-Luciferase Reporter Assay System (Promega, Madison, WI) on a luminometer (Orion II Microplate Luminometer, Berthold Detection Systems, Germany).

### Tube formation assay

Sw480 and Hct116 colorectal cancer cells were seeded in 6-well plates and incubated in RPMI 1640 supplemented with 10% FBS. Then 5.5 × 10^3^/well of HDLECs were seeded into 96-well plate which was previously painted with matrigel and 100 μl of the conditioned colorectal cancer cell mediums were added with HDLECs. Tube formation of HDLECs was observed and photographed under a microscope over 4 hours. The total numbers of tube-like structures formed in each well were measured in 3 random fields.

### Animal studies

SW480 cells were resuspended in FBS-free RPMI 1640 and were injected into 3 sites of colorectal submucosa of nude mice. The detailed process is shown in Supplementary Figure S1B. The LPS group mice were given a dose of 0.2 ml LPS (1 μg/ml) by intraperitoneal injection once every two weeks after colorectal cell plantation, while the control group got normal saline (*n* = 3 per group). After two months, the mice were anesthetized and tumors *in situ* or metastatic lymph nodes were counted and fixed in formalin.

### Immunohistochemistry

Tumors *in situ* and metastatic lymph nodes were dealt with dehydration of gradient ethanol and paraffin embedded. Then they were made into tissue sections (4 μM thick, tumor and lymph node). The sections were dewaxed in xylene and rehydrated in graded alcohol. Antigen retrieval was performed by 0.01 mol/L citrate buffer (pH6.0) for 2 minutes. Endogenous peroxidase activity was inhibited with 3% hydrogen peroxide for 10 minutes. Sections were blocked by 5% BSA for 30 minutes at room temperature, and then incubated with rabbit anti-LYVE1 (1:100; abcam, ab14917) and rabbit anti-VEGF-C (1:50; abcam, ab9546) at 4°C overnight. The following experimental procedure was according to the polink-2 plus Polymer HRP Detection System (ZSGB-bio, Beijin, China). After DAB incubation, followed was hematoxylin counter staining and covering slide. Staining results were assessed by two pathologists independently.

### Statistical analysis

Data are presented as mean and standard deviation. The two group mean differences were compared by two-sample *t*-test or paired *t*-test and analyzed by IBM SPSS statistics version 19 for Windows (IBM Corp., USA). Figures were generated by GraphPad Prism 5 (GraphPad Software, Inc., USA). A two-tailed *P* value < 0.05 was defined to be statistically significant.

## SUPPLEMENTARY MATERIALS TABLES AND FIGURE


