# Supplementary material for: Lipopolysaccharide increases the release of VEGF-C that enhances cell motility and promotes lymphangiogenesis and lymphatic metastasis through the TLR4- NF-κB/JNK pathways in colorectal cancer
Source: Oncotarget. 2016 Oct 4;7(45):73711–24. doi: 10.18632/oncotarget.12449 (PMC5342009; doi:10.18632/oncotarget.12449)
Supplement: Supplementary file 1 [file oncotarget-07-73711-s001.pdf]

Lipopolysaccharide increases the release of VEGF-C that enhances cell motility and promotes lymphangiogenesis and lymphatic metastasis through the TLR4- NF-κB/JNK pathways in colorectal cancer

Supplementary Materials

Supplementary Table S1: Standard LPS concentration (EU/ml) and absorbance (λ = 545 nm)

Standard LPS concentration	0	0.1	0.25	0.5	1
Absorbance	0	0.037	0.14	0.382	0.921

Standard curve: $y = 1.045x + 0.060$, $R^2 = 0.988$.

Supplementary Table S2: Normal and colorectal cancer tissue lysate measured absorbance (λ = 545 nm) and correspondingly calculated LPS concentration

Absorbance (A)

	1	2	3	4	5	6	7	8	9	10
NT	1.487	1.346	0.291	0.649	0.545	0.68	1.488	1.48	0.316	0.729
CC	1.496	1.595	1.635	1.564	1.545	1.571	1.529	1.625	1.593	1.593
	11	12	13	14	15	16	17	18	19	20
NT	0.839	1.243	0.858	0.935	0.864	0.785	1.074	0.782	0.453	0.865
CC	1.173	1.773	0.956	1.754	1.943	0.976	1.736	1.287	0.975	1.186

Tissue LPS concentration (EU/ml)

	1	2	3	4	5	6	7	8	9	10
NT	1.614	1.467	0.364	0.738	0.630	0.771	1.615	1.607	0.390	0.822
CC	1.624	1.727	1.769	1.695	1.675	1.702	1.658	1.758	1.725	1.725
	11	12	13	14	15	16	17	18	19	20
NT	0.937	1.359	0.957	1.037	0.963	0.881	1.183	0.877	0.534	0.964
CC	1.286	1.913	1.059	1.893	2.091	1.080	1.874	1.405	1.079	1.300

EU,endotoxin unit; NT, colorectal normal tissue; CC,colorectal cancer tissue.

Supplementary Table S3: List of oligonucleotides used in the study[a]

Oligonucleotides	Sequences (5′ 3′)	Position(nt)
Promoter cloning		
2000F	TGACTCGAGGTTGAATGGAAACTCCACCT	−2000/−1981
489F	TGACTCGAGTGCACGTGGTCCAGGGTGGT	− 489/−470
335F	TGACTCGAGCGGGGAGGAGGCGAGGGAAA	−335/−316
245F	TGACTCGAGCTCCCTCGGCCGCTTTCTCT	−245/−226
2000-245R	TGAAAGCTTCCTCCCCTTCCCCGAAGT	17/0
Real-time RT-PCR		
TLR4F	AACTCTGGATGGGGTTTCCT	
TLR4R	GTGAGACCAGAAAGCTGGGA	
VEGFR3F	TGCACGAGGTACATGCCAAC	
VEGFR3R	GCTGCTCAAAGTCTCTCACGA	
EGFCF	ATGTTTTCCTCGGATGCTGGA	
VEGFCR	CATTGGCTGGGGAAGAGTTTG	
β-actinF	CCTGGCACCCAGCACAAT	
β-actinR	GGGCCGGACTCGTCATACT	
shRNA		
shVEGFCF	GATCCCCGTCGTTGTGTCCCTTCATATTGTTC AAGAGACAATAT GAAGGGACACAACGACTTTTTA	
shVEGFCR	AGCTTAAAAAGTCGTTGTGTCCC TTCATATTGTCTCTTGAACAATAT GAAGGGACACAACGACGGG	

nt, nucleotides; RT-PCR, reverse transcription-polymerase chain reaction; shRNA, short hairpin RNA.
[a]Restriction sites are underlined.

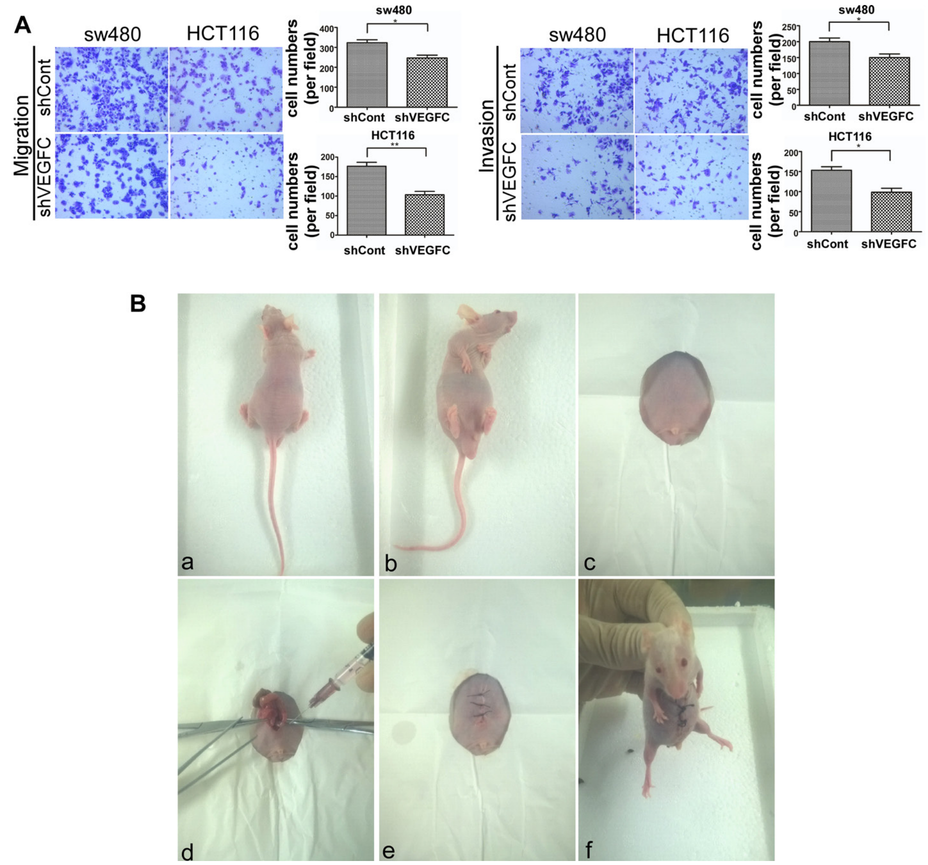

Supplementary Figure S1: (**A**) Representative images of migrated and invaded colorectal cancer cells through chambers' membrane (100×). Cell numbers were counted in three randomly selected microscopic fields. (**B**) Establishment of orthotopic xenograft model of colorectal cancer in nude mice. All mice were starved for 12 hours before model establishment. 2×10^6 sw480 colorectal cancer cells were injected into submucosa of colorectal wall mixed with matrigel after anesthesia. Then all mice were fasting for a night after operation. Error bars represent mean ± SEM, representative of three experiments, *p < %0.05, **p < %0.01.
